# PAX5 P80R-mutated B-cell acute lymphoblastic leukemia with transformation to histiocytic sarcoma: clonal evolution assessment using NGS-based immunoglobulin clonality and mutation analysis

**DOI:** 10.1007/s00428-022-03428-y

**Published:** 2022-10-14

**Authors:** Leonie I. Kroeze, B. Scheijen, K. M. Hebeda, J. Rijntjes, J. A. C. W. Luijks, D. Evers, W. Hobo, P. J. T. A. Groenen, M. van den Brand

**Affiliations:** 1 Department of Pathology, Radboud University Medical Center, Geert Grooteplein Zuid 10, 6525GA Nijmegen, the Netherlands; 2 Radboud Institute for Molecular Life Sciences, Nijmegen, the Netherlands; 3 Department of Hematology, Radboud University Medical Center, Nijmegen, the Netherlands; 4 Department of Laboratory Medicine - Laboratory of Hematology, Radboud University Medical Center, Nijmegen, the Netherlands

**Keywords:** Acute lymphoblastic leukemia, Histiocytic sarcoma, Clonal evolution, Clonality analysis, Transformation

## Abstract

Clonality assessment by the detection of immunoglobulin (IG) gene rearrangements is an important method to determine whether two concurrent or subsequent lymphoid malignancies in one patient are clonally related. Here, we report the detailed clonality analysis in a patient with a diagnosis of B-cell acute lymphoblastic leukemia (B-ALL) followed by a histiocytic sarcoma (HS), in which we were able to study clonal evolution by applying next generation sequencing (NGS) to identify IG rearrangements and gene mutations. Using the sequence information of the NGS-based IG clonality analysis, multiple related subclones could be distinguished in the PAX5 P80R-mutated B-ALL. Notably, only one of these subclones evolved into HS after acquiring a *RAF1* mutation. This case demonstrates that NGS-based IG clonality assessment and mutation analysis provide clear added value for clonal comparison and thereby improves clinicobiological understanding.

**Supplementary Information:**

The online version contains supplementary material available at 10.1007/s00428-022-03428-y.

## Case introduction

**Fig. 1:**
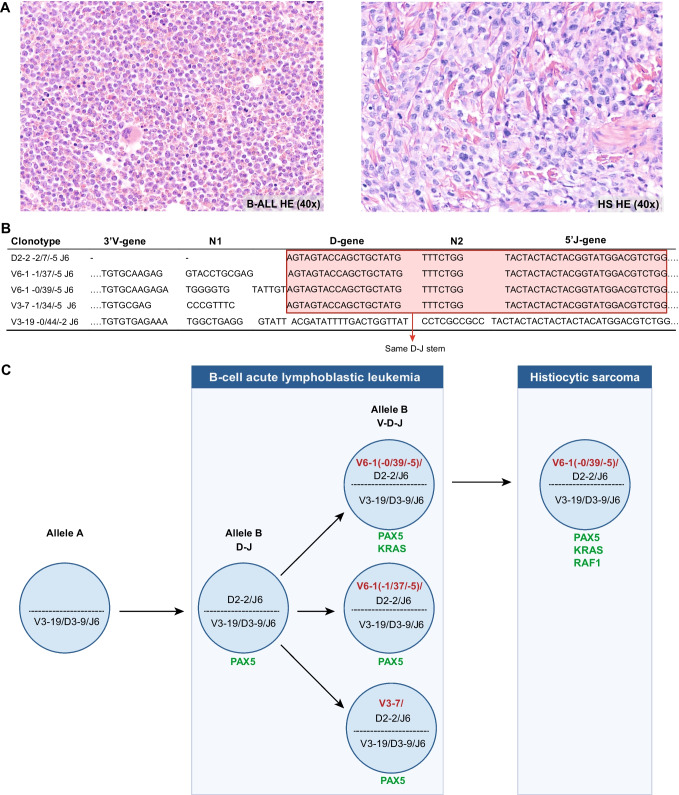
Clonality assessment of B-cell acute lymphoblastic leukemia (B-ALL) and histiocytic sarcoma (HS). **A** Immunophenotyping. The H&E staining (40 × magnification) of the B-ALL in the bone marrow biopsy shows a monomorphic proliferation of small blasts. The H&E staining of the HS in the skin shows a proliferation of pleiomorphic cells with abundant eosinophilic cytoplasm. Additional immunoflowcytometry and immunohistochemistry data are listed in the supplementary Tables 1 and 3. **B** Comparison of IGH rearrangements in the B-ALL sample. The red square highlights the identical IGHD-IGHJ sequences (D-J stem). N indicates added nucleotides at the junction. **C** Clonal evolution based on IGH rearrangements and mutations. Based on the identified IGH rearrangements, it seems that first an IGHV-IGHD-IGHJ rearrangement occurred on one IGH allele (allele A). Subsequently, the other IGH allele (allele B) started to rearrange by combining an IGHD and IGHJ gene after which a catastrophic event occurred, leading to several subclones in which a different V gene (indicated in red; for the two subclones containing IGHV6-1 the difference in the junction is indicated as well) was joined to the existing IGHD-IGHJ. One of these subclones evolved into HS, supported by the detection of two 100% identical IGH rearrangements (and one identical IGK rearrangement) in both samples. Also, the identified mutations supported a clonal relationship (relevant mutated genes are shown in green). The *PAX5* mutation is likely an early event after which additional mutations occurred in the different subclones. The *KRAS* mutated clone evolved into histiocytic sarcoma. A novel *RAF1* mutation might have played a role in the switch from B-ALL to HS

A 60-year-old male patient presented with fatigue. Analysis of the peripheral blood showed pancytopenia and 40% of blasts. Subsequent analysis of a bone marrow biopsy and aspirate resulted in a diagnosis of BCR-ABL-negative B-cell acute lymphoblastic leukemia (B-ALL, for immunophenotype and karyotype, see supplementary Tables 1 and 2), with absence of extramedullary and central nervous system localization. The patient started intensive chemotherapy and rapidly reached complete remission with no detectable minimal residual disease (MRD) by cytomorphology and immunophenotyping. However, 10 months after the initial diagnosis of B-ALL and during consolidation courses, the patient presented with recurrent thrombocytopenia, progressive fatigue and a skin lesion on the forehead. A positron emission tomography (PET) scan showed generalized disease with foci of increased FDG uptake in bone, lymph nodes, the liver, the skin, and the lungs. Morphology and immunohistochemistry of biopsies obtained from the bone marrow, skin, and a supraclavicular lymph node showed an infiltration of rather large, pleomorphic malignant cells (Fig. 1A) with a high proliferation (Ki-67: 80%) and histiocytic differentiation (CD14^+^, CD68^+^, and CD163^+^, supplementary Table 3), consistent with a diagnosis of histiocytic sarcoma (HS). The biopsies, peripheral blood, and bone marrow aspirate did not demonstrate any evidence of B-ALL relapse. To determine whether the two malignancies in this patient were clonally related, we performed clonality assessment by using GeneScan and NGS-based immunoglobulin (IG) clonality analysis and mutation analysis.

## Methods

### IG clonality analysis using GeneScan (EuroClonality/BIOMED-2 assay)

BIOMED-2 multiplex PCRs and GeneScan analysis were performed according to standard procedures [1]. For each target, a duplicate analysis was performed with 20 and 40 ng DNA input.

### IG clonality analysis using NGS

PCR library preparation and sequencing were performed as described previously [2]. 40 ng DNA was used as input for the library preparation. Sequencing data were visualized and analysed using the ARResT/Interrogate software platform.

### Trusight oncology 500

Library preparation was performed using the hybrid capture-based TruSight Oncology 500 Library Preparation Kit (TSO500; Illumina) following the manufacturer’s protocol. 60 ng DNA was used as input for the library preparation. Sequencing and data analysis were performed as described previously [3].

## Results

To determine whether the B-ALL and HS had a common clonal origin, we performed immunoglobulin (IG) clonality analysis on a bone marrow sample at the time of B-ALL diagnosis and a skin biopsy with HS infiltration. With conventional clonality analysis using GeneScan fragment length analysis according to the EuroClonality/BIOMED-2 assay[1], IGHV-IGHD-IGHJ rearrangement analysis showed similarly sized products for framework (FR)1, FR2, and FR3 targets in both the B-ALL and HS sample, but also products that were unique for the B-ALL or HS sample were observed (Table 1, Supplementary Fig. 1). For IGHD-IGHJ, IGKV-IGKJ, and IGKV/Intron-KDE targets, only clonal products unique for one of the two samples could be detected. This analysis was therefore not firmly conclusive to establish whether the B-ALL and HS were clonally related, since only one IGHV-IGHD-IGHJ gene rearrangement (as convincingly detected in the FR1 and FR2 PCRs) showed an identically sized fragment, which could be a coincidence. In addition, the results of the B-ALL sample suggested the presence of more than one clone based on the number of IGH gene rearrangements.

**Table 1:** IG clonality: GeneScan and NGS results

IG locus	B-cell acute lymphoblastic leukemia			Histiocytic sarcoma		
	GS results	NGS results		GS results	NGS results	
	Peak size	Clonotype	Frequency	Peak size	Clonotype	Frequency
IGHV-IGHJ FR1	C364bp	n.a	n.a	C364bp	n.a	n.a
	Cw362bp	n.a	n.a	-	n.a	n.a
	Cw349bp	n.a	n.a	-	n.a	n.a
IGHV-IGHJ FR2	C299bp	n.a	n.a	C299bp	n.a	n.a
	C284bp	n.a	n.a	-	n.a	n.a
	C292bp	n.a	n.a	-	n.a	n.a
	-	n.a	n.a	C295bp	n.a	n.a
IGHV-IGHJ FR3	Cw164bp	V6-1 -0/39/-5 J6	5%	C164bp	V6-1 -0/39/-5 J6	65%
	C149bp	V3-7 -1/34/-5 J6	17%	-	-	-
	Cw161bp	V6-1 -1/37/-5 J6	15%	-	-	-
	-	V3-19 -0/44/-2 J6	46%	-	V3-19 -0/44/-2 J6	31%
IGHD-IGHJ	C257bp	D2-2 -2/7/-5 J6	97%	nsp	nsp	-
IGKV-IGKJ	Cw135bp	V1(D)-33 -11/2/-7 J3	64%	-	V1(D)-33 -11/2/-7 J3	25%
	-	-	-	C293bp	V2D-26 -1/2/-0 J4	74%
IGKV/intron-IGKde	C292bp	intron -0/8/-0 Kde	11%	nsp	nsp	-

Remark: Since different primers are used for GS and NGS, some rearrangements might have been missed/underrepresented with one of the techniques because of suboptimal primer annealing. An example is the IGHV-IGHD-IGHJ V3-19 -0/44/-2 J6 clonotype, which cannot be detected by GS because of alterations at the 3′end of the BIOMED2 primer binding site. A clonotype is defined as a rearrangement that is denoted as a combination of the 5′ gene, the number of deleted and added nucleotides at the junction and the 3′ gene

n.a. indicates not applicable (FR1 and FR2 targets were not analyzed by NGS); scoring: *nsp*, no specific products; *C*, clonal; *Cw*, clonal weak; *GS*, GeneScan; *NGS*, next-generation sequencing; and *FR*, framework. Underlined indicates rearrangements identified in both samples

To more reliably determine the clonal relationship between the B-ALL and HS in this patient, we performed NGS-based clonality analysis, according to the protocol that was recently published by the EuroClonality-NGS Working Group [2, 4]. With NGS-based IG clonality analysis, two clonal IGHV-IGHD-IGHJ rearrangements (clonotypes V6-1 -0/39/-5 J6 and V3-19 -0/44/-2 J6) and one clonal IGKV-IGKJ rearrangement (V1(D)-33 -11/2/-7 J3) were detected in both the B-ALL and HS with 100% identical sequences, providing evidence for a direct clonal relationship (Table 1, Supplementary Fig. 2). Nonetheless, additional IG rearrangements unique for each sample were also detected. Detailed analysis of the sequences of the five IGH rearrangements in the B-ALL sample revealed that four of the rearrangements, i.e., one incomplete IGHD-IGHJ and three complete IGHV-IGHD-IGHJ rearrangements, contained the exact same D and J genes, including an identical N2 junction (D-J stem: D2-2 -2/7/-5 J6). Two different V genes were combined with this IGHD-IGHJ stem, resulting in three different clonotypes (V6-1 -1/37/-5 J6, V6-1 -0/39/-5 J6 and V3-7 -1/34/-5 J6) (Fig. 1B). This suggests that (at least) three subclones in the B-ALL arose from the same IGHD-IGHJ stem. Based on the rearrangements detected in the HS sample, only one of the subclones evolved into the HS (Fig. 1C).

To further study the pattern of clonal evolution from the B-ALL (sub)clone to HS, molecular analysis was performed with the TruSight Oncology 500 assay (Illumina), which identified multiple mutations and several copy number variations in both the B-ALL and HS sample (Supplementary Tables 4 and 5). A pathogenic mutation in the *PAX5* gene (resulting in the p.P80R change) was detected indicating that the B-ALL belongs to the newly recognized subtype of B lymphoblastic leukemia with PAX5 P80R [5, 6]. In addition, mono-allelic loss of *PAX5* was observed, leading to bi-allelic inactivation of the *PAX5* gene. These *PAX5* alterations, together with a mutation in *KRAS* (p.G12D) and loss of *CDKN2A*, were identified in both the B-ALL and HS tissue, which supported the clonal relationship. An additional, likely oncogenic *RAF1* (p.R391W) mutation, was detected in the HS sample only [7].

## Discussion

Lineage switch of a lymphoid malignancy to HS is rare, but has been well documented in previous case reports and in small case series [8–11]. A clonal relationship between the lymphoid malignancy and the HS was established in a subset of cases by the detection of identical cytogenetic abnormalities or rearrangements of the immunoglobulin or T-cell receptor genes. In our case, conventional clonality analysis yielded ambiguous results, but NGS-based IG clonality analysis was able to confirm the clonal relationship between the B-ALL and the HS. In addition, using the sequence information of the NGS-based IG clonality analysis, multiple related subclones could be distinguished in the B-ALL, and the clonal evolution to HS could be unraveled (Fig. 1C). The clonal relationship was further supported by the presence of identical somatic mutations in *PAX5* and *KRAS*.

The reason why some B-ALL patients develop a histiocytic malignancy is not clear, yet. Several studies show that loss of PAX5 expression in B-cells leads to dedifferentiation to uncommitted precursor cells [12, 13]. Interestingly, the present B-ALL case displayed inactivated *PAX5* due to a mutation combined with loss of the other *PAX5* allele, which will result in a lack of PAX5 expression. This likely makes these cells prone to loss of their B-cell phenotype and differentiate into another lineage after acquiring one or more additional genetic aberrations. Furthermore, specific aberrations in B-ALL, including PAX5-P80R, were recently found to predispose to an early monocytic phenotype switch in pediatric patients during initial chemotherapy [14]. The monocytic switch was accompanied by a gradual loss of CD19 expression and increase in expression of at least one monocytic marker. This observation also shows that *PAX5*-mutated cells are able to rather easily switch their phenotype. In this pediatric PAX5-P80R-mutated cohort, no subsequent malignancies, like histiocytic sarcoma, were described during follow-up.

In our adult case, the B-ALL cells harbored a *PAX5* mutation and subclonal *KRAS, NRAS, PTPN11*, and *CDKN2A* mutations. In addition, mono-allelic loss of *PAX5* and *CDKN2A* was detected. All these aberrations are frequently observed in *PAX5*-mutated B-ALL patients. Passet et al. [5] described a cohort of 30 PAX5-P80R-mutated adult B-ALL patients which all showed inactivation of the second *PAX5* allele (mutation or loss) and the majority harbored *CDKN2A* loss (74%) and an *NRAS* and/or *KRAS* mutation (73%). All patients achieved complete remission after treatment. Monocytic shift or subsequent histiocytic malignancies were not reported in this paper.

Interestingly, the HS cells of our patient harbored the same *PAX5* and *KRAS* mutation as the B-ALL cells, with an additional *RAF1* mutation. The combinaton of multiple mutations effecting the RAS pathway has been reported before in HS, including the combination of a *KRAS* with a *RAF1* mutation [9, 15]. Also in one of these previously described cases, the *RAF1* mutation was present only in the HS sample and not in the concomitant chronic myelomonocytic leukemia (CMML), whereas both harbored the same *KRAS* mutation [9]. These observations suggest that RAF1 activation might play a role in the final transformation to HS in B-cells that are already prone to loss of their B-cell phenotype.

In conclusion, we report a case of PAX5 P80R-mutated B-ALL followed by histiocytic sarcoma in which combined NGS-based IG clonality and mutational analysis elucidated the clonal relationship and evolution. A combination of genetic events, including *PAX5* inactivation in combination with an acquired *RAF1* mutation, may have played a role in the lineage switch of the malignant cells and subsequent development of HS. Our study demonstrates that NGS-based mutation and IG clonality analysis provide clear added value for clonal comparison, which helps to unravel the underlying clinicopathological mechanisms of disease evolution.

## Supplementary Information

Below is the link to the electronic supplementary material.

Supplementary file1 (PDF 1317 KB)
