# Supplementary material for: PAX5 P80R-mutated B-cell acute lymphoblastic leukemia with transformation to histiocytic sarcoma: clonal evolution assessment using NGS-based immunoglobulin clonality and mutation analysis
Source: Virchows Arch. 2022 Oct 14;483(1):105–10. doi: 10.1007/s00428-022-03428-y (PMC10326092; doi:10.1007/s00428-022-03428-y)
Supplement: Supplementary file 1 — Supplementary file1 (PDF 1317 KB) [file 428_2022_3428_MOESM1_ESM.pdf]

Supplementary information

Title

PAX5 P80R-mutated B-cell acute lymphoblastic leukemia with transformation to histiocytic sarcoma – clonal evolution assessment using NGS-based immunoglobulin clonality and mutation analysis

Journal:

Virchows Archiv

Authors:

L. I. Kroeze[1], B. Scheijen[1,2], K.M. Hebeda[1], J. Rijntjes[1], J.A.C.W. Luijks[1], D. Evers[3], W.Hobo[4], P.J.T.A. Groenen[1], M. van den Brand[1]

Affiliations:

[1]Department of Pathology, Radboud university medical center, Nijmegen, The Netherlands

[2]Radboud Institute for Molecular Life Sciences, Nijmegen, The Netherlands

[3]Department of Hematology, Radboud university medical center, Nijmegen, The Netherlands

[4]Department of Laboratory Medicine - Laboratory of Hematology, Radboud university medical center, Nijmegen, The Netherlands.

Corresponding author:

Leonie I. Kroeze

leonie.kroeze@radboudumc.nl

ORCID-ID: 0000-0002-2666-1742

Supplementary Tables

Supplementary Table 1: Immunoflowcytometric phenotyping of the blasts in the bone marrow discriminated 2 B-cell precursor populations, that showed both expression of CD19, CD22, CD34, cytoplasmic CD179a and TdT, but the expression levels and some additional markers (CD10) differed.

Population	CD19	CD34	CD33	CD10	CD20	HLADR	CD38	cytCD79a+	cytCD179a+	TdT	CD22
1	+	+	+ (50%)	+ (22%)	-	+	+	+	+	+	+ (55%)
2	++	+	+	-	-	++	++	+	+	+	+

Supplementary Table 2: SNP-array

Array results	1q21.1qter(143932350_249224684)x3[0.35]	[gain 105,3 Mb]
	9pterp13.1(192129_38787480)x1	[deletie 38,6 Mb; bevat CDKN2A/B en PAX5 gen]
	16q11.2qter(46463673_90163275)x1[0.35]	[deletie 43,7 Mb]
	20q11.21q13.2(31532616_50232497)x1	[deletie 18,7 Mb]
	20q13.2qter(54937543_62915556)x1	[deletie 8 Mb]
Array-based karyotype	46,XY,dup(1)(q21.1qter),del(9)(pterp13.1),del(16)(q11.2qter),del(20)(q11.21q13.2),del(20)(q13.2qter)	

Supplementary Table 3: Immunohistochemical expression of the histiocytic sarcoma in the skin.

Positive markers	Negative markers
CD68	CD20
CD163	CD79a
CD14	CD10
CD4 (weak)	CD34
Lysozyme (partly)	TdT
	CD117
	MPO
	CD1a
	CD123
	CD23
	CD56
	TCL-1
	Tryptase

Supplementary Table 4: Pathogenic mutations detected using TruSight oncology 500

			**B-ALL (90% tumor cells)**	**HS (70% tumor cells)**
**Gene**	**Transcript**	**Mutation**	**VAF**	**VAF**
PAX5	NM_016734.2	c.239C>G p.(Pro80Arg)	88%	56%
KRAS	NM_004985.5	c.35G>A p.(Gly12Asp)	4%	31%
CDKN2A	NM_000077.4	c.151dup p.(Val51fs)	14%	-
PTPN11^	NM_002834.5	c.218C>T p.(Thr73Ile)	14%	-
PTPN11#	NM_002834.5	c.1508G>C p.(Gly503Ala)	9%	-
PTPN11#	NM_002834.5	c.1507G>A p.(Gly503Arg)	3%	-
PTPN11#	NM_002834.5	c.1504T>C p.(Ser502Pro)	3%	-
PTPN11^	NM_002834.5	c.215C>T p.(Ala72Val)	2%	-
NRAS	NM_002524.5	c.37G>C p.(Gly13Arg)	1%	-
RAF1	NM_002880.3	c.1171A>T p.(Arg391Trp)	- *	37%
Median unique coverage			737x	807x

* Covered by 932 unique reads: not any T detected at this position in the B-ALL sample

# and ^: mutations close together, but present in different sequencing reads

Supplementary Table 5: Copy number variations detected using TruSight oncology 500

	**B-ALL (90% tumor cells)**	**HS (70% tumor cells)**
**CDKN2A**	mono-allelic loss*	bi-allelic loss
**PAX5**	mono-allelic loss	mono-allelic loss

* It can not be excluded that bi-allelic loss is present in a small subclone

Supplementary Figures

A: IGHV-IGHJ FR1

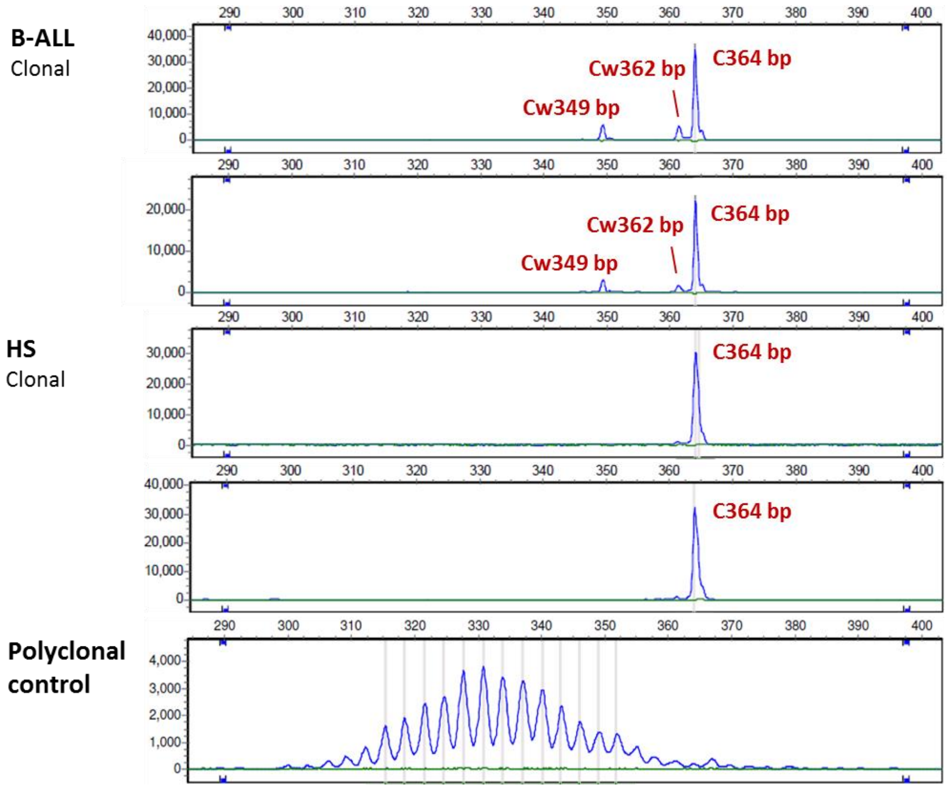

B: IGHV-IGHJ FR2

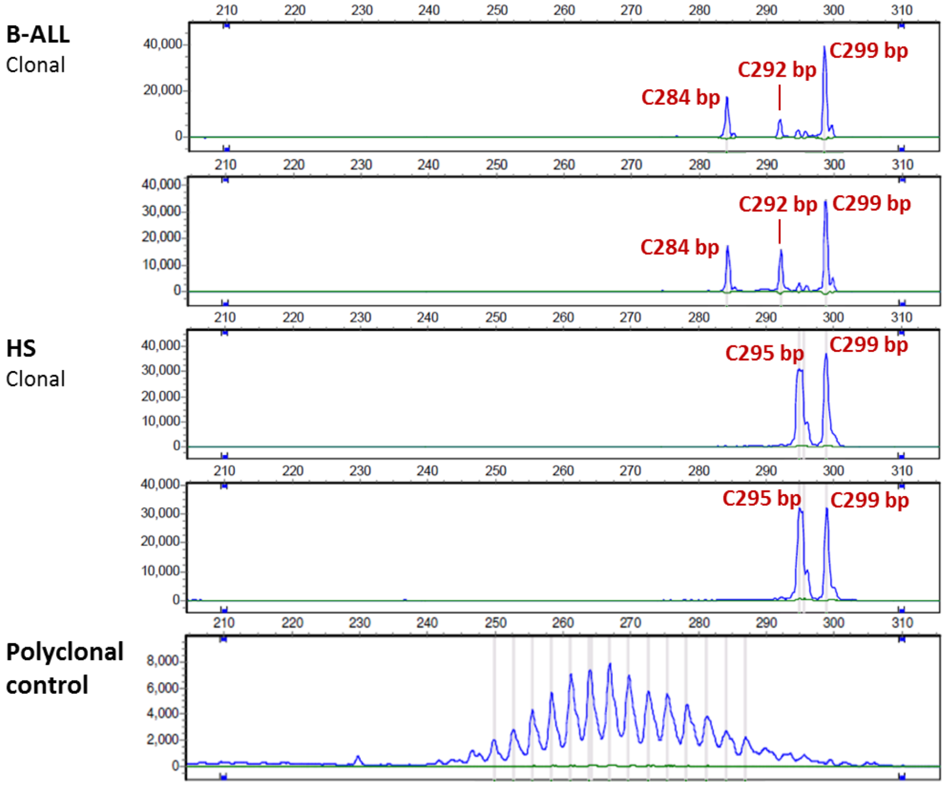

C: IGHV-IGHJ FR3

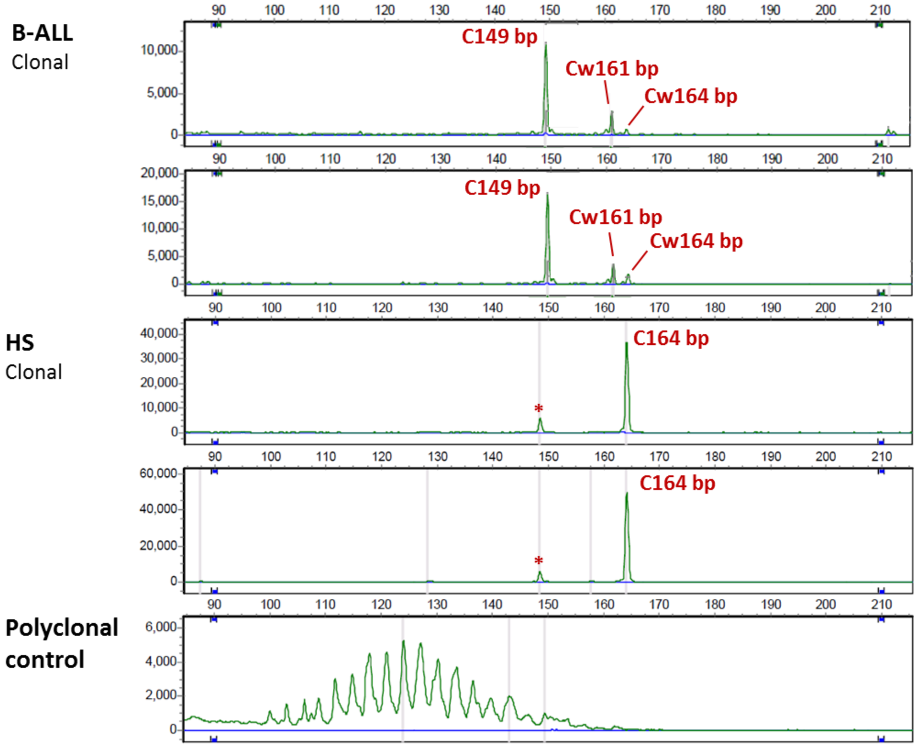

D: IGHD-IGHJ

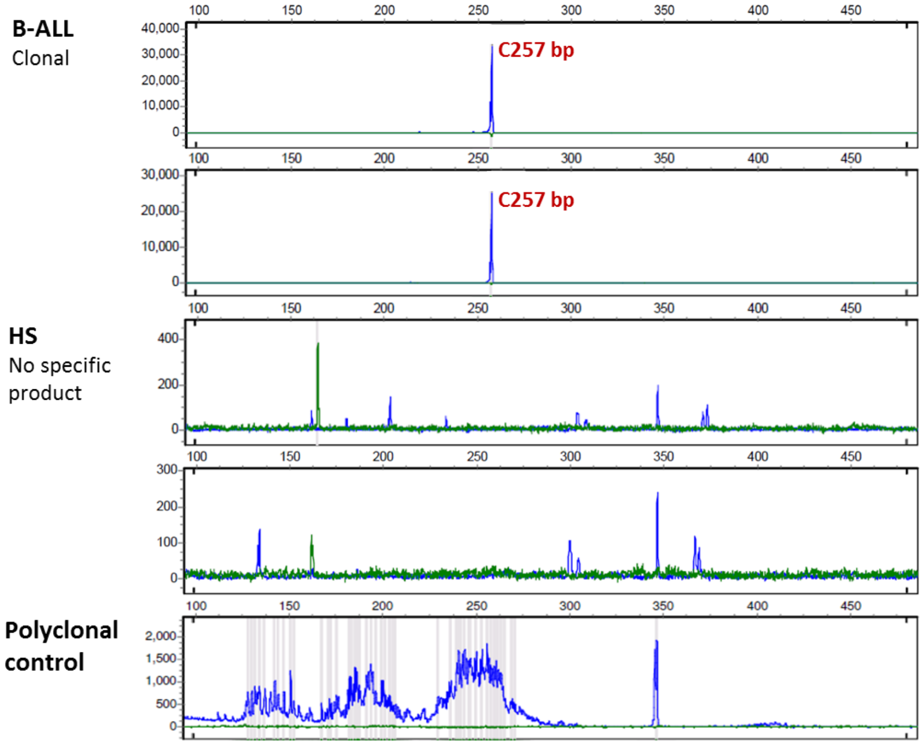

E: IGKV-IGKJ

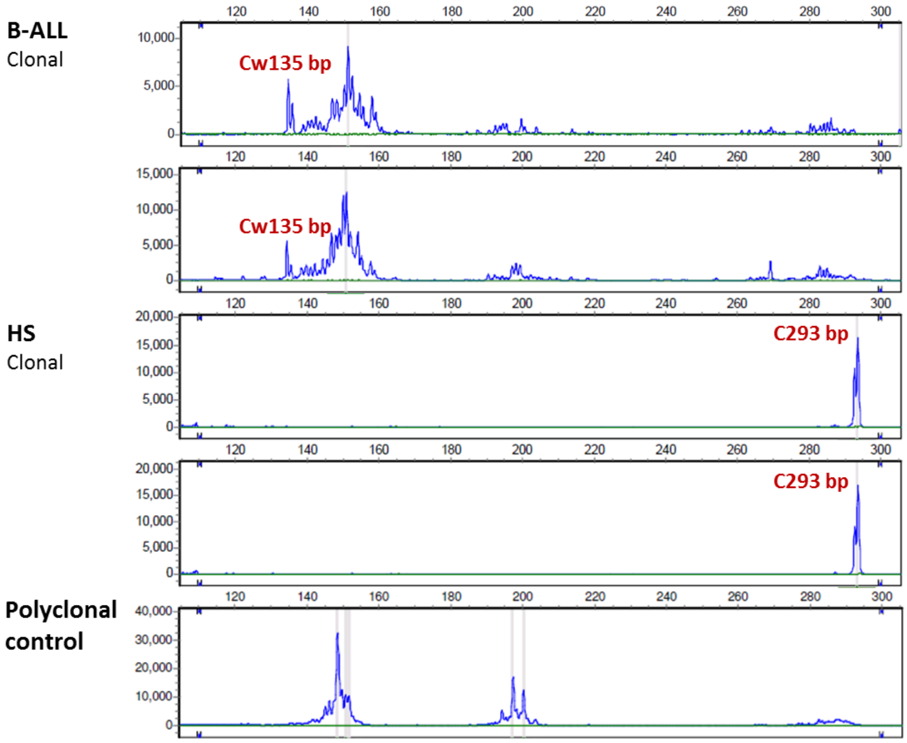

F: IGKV/intron-IGKde

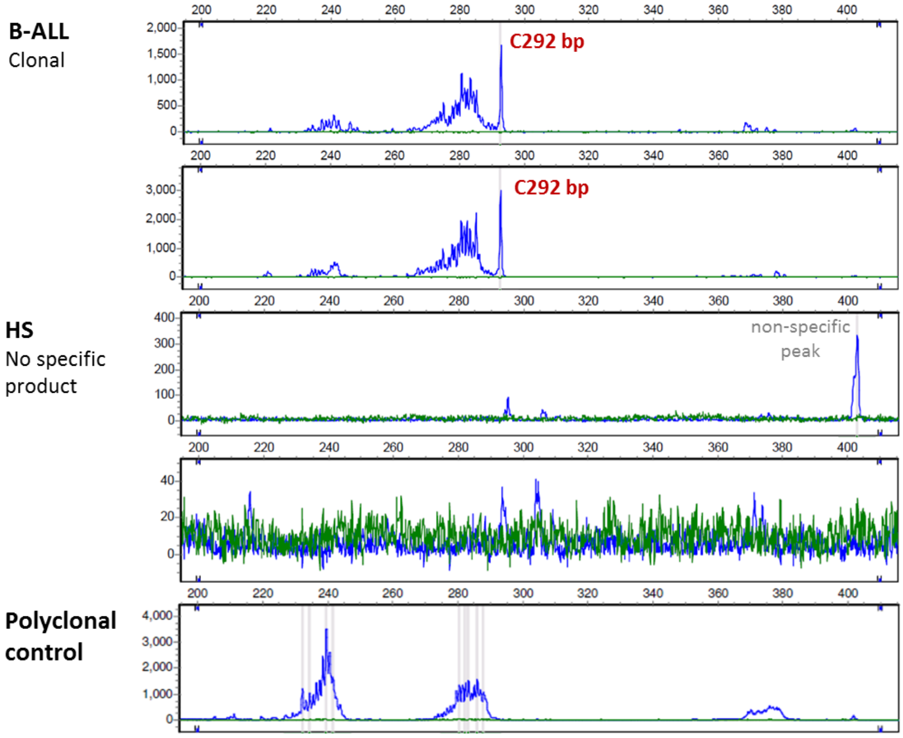

Supplementary Fig. 1: GeneScanning results of the EuroClonality/BIOMED-2 IG assays. For each target a duplicate analysis for the ALL and the HS sample is shown and in addition a polyclonal control. A) IGHV-IGHJ FR1; B) IGHV-IGHJ FR2; C) IGHV-IGHJ FR3; D) IGHD-IGHJ; E) IGKV-IGKJ; F) IGKV/intron-IGKde. B-ALL, B-cell acute lymphoblastic leukemia; HS, histiocytic sarcoma; FR, framework; C, clonal; Cw, clonal weak; and bp, base pairs.

* Sanger sequencing analysis of this product (Cw148 bp) showed that this is a result of cross-annealing of the IGH V4 primer to the V6-1 gene. Therefore, this small peak also represents the V6-1 rearrangement (similar as the C164 bp product resulting from the IGH V6 primer).

A: IGHV-IGHJ FR3

B-ALL: Clonal (122028 reads)

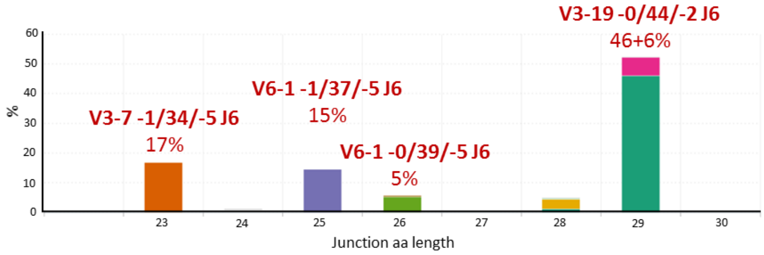

rearrangement type	clonotype	junction in amino acids	junction information	junction aa length	%
VJ:Vh-(Dh)-Jh	V3-19 -0/44/-2 J6	CVRNG*GYYDILTGYPRR#YYYYYYMDVW	unp	29	46.2
VJ:Vh-(Dh)-Jh	V3-7 -1/34/-5 J6	CASPFQ*YQLLCFWYYYYGMDVW	unp	23	16.9
VJ:Vh-(Dh)-Jh	V6-1 -1/37/-5 J6	CARGTCESSTSCYVS#YYYYGMDVW	unp	25	14.5
VJ:Vh-(Dh)-Jh	V3-19 -0/45/02 J6	CVRNG*GY*RYFDWLSSP#YYYYYYMDVW	unp	29	6.2
VJ:Vh-(Dh)-Jh	V6-1 -0/39/-5 J6	CARDGVYCSSTSCYVS#YYYYGMDVW	unp	26	5.3
VJ:Vh-(Dh)-Jh	V3-19 -0/43/-4 J6	CVRNG*GYYDILTGYPRR#YYYYYMDVW	unp	28	3.3
VJ:Vh-(Dh)-Jh	V3-19 -0/44/-2 J6	CVRNG*GYYDILTGYPRR#YYYYYYMDVW	unp	28	1.2
VJ:Vh-(Dh)-Jh	V3-7 -1/43/-5 J6	CARVPF*L**YQLLCFWYYYYGMDVW	unp	26	0.4
VJ:Vh-(Dh)-Jh	V3-7 -0/39/-5 J6	CARRG*L**YQLLCFWYYYYGMDVW	unp	25	0.2
VJ:Vh-(Dh)-Jh	V1-2 -0/44/-5 J6	CAREETKDIVVVPAAMFL#YYYYGMDVW	unp	28	0.2

HS: Clonal (105422 reads)

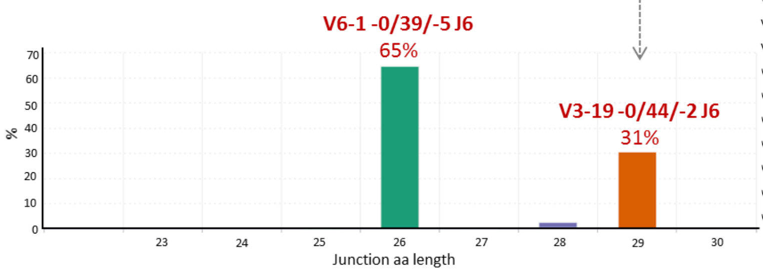

rearrangement type	clonotype	junction in amino acids	junction information	junction aa length	%
VJ:Vh-(Dh)-Jh	V6-1 -0/39/-5 J6	CARDGVYCSSTSCYVS#YYYYGMDVW	unp	26	64.6
VJ:Vh-(Dh)-Jh	V3-19 -0/44/-2 J6	CVRNG*GYYDILTGYPRR#YYYYYYMDVW	unp	29	30.6
VJ:Vh-(Dh)-Jh	V3-19 -0/43/-4 J6	CVRNG*GYYDILTGYPRR#YYYYYMDVW	unp	28	1.8
VJ:Vh-(Dh)-Jh	V3-19 -0/44/-2 J6	CVRNG*GYYDILTGYPRR#YYYYYYMDVW	unp	28	0.8
VJ:Vh-(Dh)-Jh	V6-1 -0/32/-1 J6	CARDGVYCSSTSCY#FWYYYGMDVW	unp	25	0.4
VJ:Vh-(Dh)-Jh	V6-1 -0/39/-5 J6	CARDGVYCSSTSCYVS#YYYYGMDVW	unp	25	0.1
VJ:Vh-(Dh)-Jh	V3-19 -0/39/-5 J6	CARDGVYCSSTSCYVS#YYYYGMDVW	unp	26	0.1
VJ:Vh-(Dh)-Jh	V3-19 -0/47/-2 J6	CVRNG*GYYDILTGYPRRL#YYYYYYMDVW	unp	30	0.08
VJ:Vh-(Dh)-Jh	V3-19 -0/42/-2 J6	CVRNG*GITIFDWLSSP#YYYYYYMDVW	unp	28	0.07
VJ:Vh-(Dh)-Jh	V6-1 -0/39/-5 J6	CAREWVYCSSTSCYVS#YYYYGMDVW	unp	26	0.07

B: IGHD-IGHJ

B-ALL: Clonal (24106 reads)

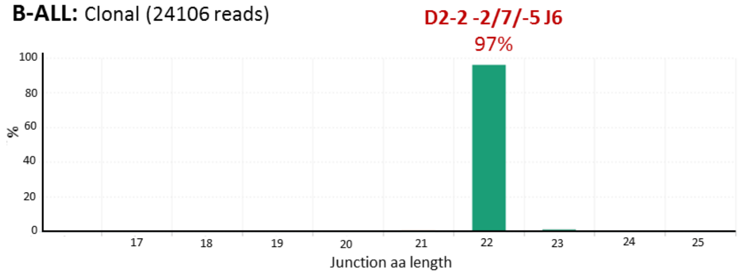

rearrangement type	clonotype	junction in amino acids	junction information	junction aa length	%
DJ:Dh-Jh	D2-2 -2/7/-5 J6	VRIL**YQLLCFWYYYYGMDVW	pop	22	97.2
DJ:Dh-Jh	D2-2 -2/7/-5 J6	VRIL**YQLLCFWYYYYGMDVW	pop	23	1.1
DJ:Dh-Jh	D2-2 -2/0/-1 J6	VRIL**YQLLCFWYYYGMDVW	pop	21	0.8
DJ:Dh-Jh	D2-2 -2/12/-8 J6	VRIL**YQLLCFWL#YYYGMDVW	pop	23	0.07
DJ:Dh-Jh	D2-2 -2/2/-2 J5	VRIL**YQLLW#NWFDPW	pop	18	0.06
DJ:Dh-Jh	D5-5=D5-18 -1/12/-3 J4	VVDTAMVRGTAHFDYW	pop	16	0.05
DJ:Dh-Jh	D2-2 -2/7/-5 J6	VRIL**YQLLCFW#LLLRYGTSW	pop	23	0.05
DJ:Dh-Jh	D2-2 -2/7/-5 J6	VRIL**YQLLCFWYYYYGMDVR	pop	22	0.03
DJ:Dh-Jh	D2-2 -2/7/-2 J6	VRIL**YQLLCFWYYYYYGMDVW	pop	23	0.03
DJ:Dh-Jh	D1-26 -3/11/-5 J4	VGIVGATPGV#FDYW	pop	15	0.03

HS: No specific product (5 reads)

C: IGKV-IGKJ

B-ALL: Clonal (89337 reads)

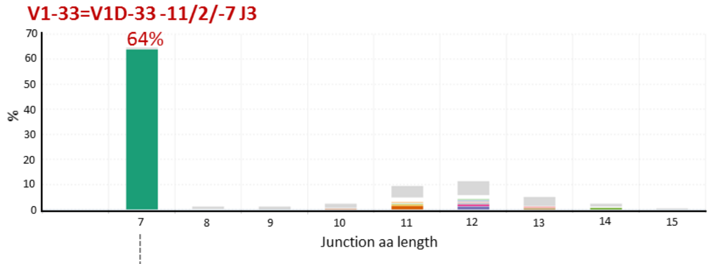

rearrangement type	clonotype	junction in amino acids	junction information	junction aa length	%
VJ:Vk-Jk	V1-33=V1D-33 -11/2/-7 J3	CQQYD#F	unp	7	64.1
VJ:Vk-Jk	V2-30=V2D-30 -0/3/-7 J3	CMQGTHWPP#F	unp	11	1.4
VJ:Vk-Jk	V1-33=V1D-33 -0/0/-1 J4	CQQYDNLP#LTF	unp	12	1.3
VJ:Vk-Jk	V4-1 -0/0/-0 J4	CQQYYSTPPLTF	pro	12	0.8
VJ:Vk-Jk	V1-39=V1D-39 -3/9/-0 J4	CQQSYSTPGGSLTF	pro	14	0.7
VJ:Vk-Jk	V1-37=V1D-37 -0/0/-5 J4	GQRTYNAP##F	unp	11	0.6
VJ:Vk-Jk	V1-33=V1D-33 -1/10/-6 J4	CQQYDNLPLKAHF	pro	13	0.5
VJ:Vk-Jk	V4-1 -5/2/-0 J2	CQQYYST##CSF	unp	12	0.4
VJ:Vk-Jk	V4-1 -1/0/-4 J4	CQQYYSTP#TF	unp	11	0.4
VJ:Vk-Jk	V1-37=V1D-37 -2/1/-0 J4	GQRTYNAP#LTF	unp	12	0.4

HS: Clonal (124755 reads)

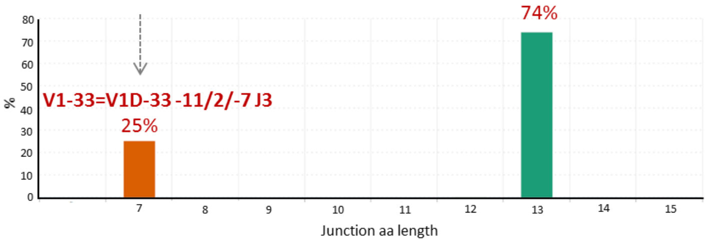

rearrangement type	clonotype	junction in amino acids	junction information	junction aa length	%
VJ:Vk-Jk	V2D-26 -1/2/-0 J4	CMQDAQDPQ#LTF	unp	13	74.1
VJ:Vk-Jk	V1-33=V1D-33 -11/2/-7 J3	CQQYD#F	unp	7	25.3
VJ:Vk-Jk	V2D-26 -1/2/-0 J4	CMQDAQDPQ#LTF	unp	12	0.2
VJ:Vk-Jk	V2D-26 -1/2/-0 J4	CMARCTRSSGSLF	pro	13	0.03
VJ:Vk-Jk	V2D-26 -1/2/-0 J4	CMARCTRSS#LTF	unp	13	0.02
VJ:Vk-Jk	V1-33=V1D-33 -11/2/-7 J3	CQQYD#F	unp	6	0.02
VJ:Vk-Jk	V2D-26 -1/2/-0 J4	RMARCTRSS#LTF	unp	13	0.02

D: IGKV/intron-IGKde

B-ALL: Clonal (11232 reads)

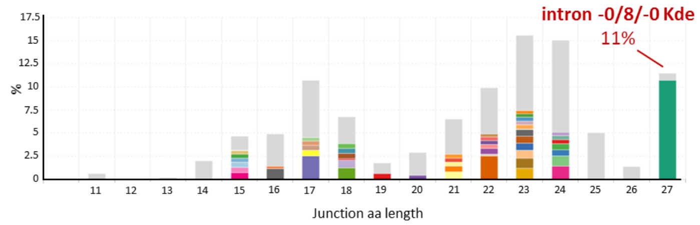

rearrangement type	clonotype	junction in amino acids	junction information	junction aa length	%
intron-Kde	intron -0/8/-0 Kde	PCVCPIDAAVASFPDASN#SPSGSPGR	unk	27	10.7
Vk-Kde	V1-37=V1D-37 -4/3/-0 Kde	GQRTYNALRSPSGSPGR	unk	17	2.5
intron-Kde	intron -8/2/-2 Kde	PCVCPINAAVASF#SPSGSPGR	unk	22	2.5
intron-Kde	intron -3/3/-0 Kde	PCVCPINAAVASFPGRSPSGSPGR	unk	24	1.4
intron-Kde	intron -3/4/-20 Kde	PCVCPIDAAVASFPG#GR	unk	18	1.2
intron-Kde	intron -2/0/-3 Kde	PCVCPINAAVASFP##PSGSPGR	unk	23	1.2
Vk-Kde	V4-1 -10/4/-0 Kde	CQQYYT##SPSGSPGR	unk	16	1.1
intron-Kde	intron -2/0/-3 Kde	PCVCPIDAAVASFP##PSGSPGR	unk	23	1.1
intron-Kde	intron -0/0/-2 Kde	PCVCPIDAAVASFPD#SPSGSPGR	unk	24	1.1
Vk-Kde	V5-2 -2/10/-6 Kde	CLQHDNFPSSSSSGSPGR	unk	18	0.9

HS: No specific product (2 reads)

Supplementary Fig. 2: NGS-based clonality results. For each target the result from the B-ALL and HS sample are shown. On the left the top 50 most abundant clonotypes are visualised in colour, based on the amino acid (AA) junction length (X-axis) and frequency (Y-axis). On the right the top 10 most frequent clonotypes are shown in a table format. A) IGHV-IGHJ FR3; B) IGHD-IGHJ; C) IGKV-IGKJ; D) IGKV/intron-IGKde. B-ALL, B-cell acute lymphoblastic leukemia; HS, histiocytic sarcoma; FR, framework; unp, unproductive; pro, productive; pop, potentially productive; and unk, unknown.
